# Copper deficiency and effects of copper supplementation in a herd of red deer (*Cervus elaphus*)

**DOI:** 10.1186/1751-0147-50-8

**Published:** 2008-04-30

**Authors:** Kjell Handeland, Aksel Bernhoft, Magne S Aartun

**Affiliations:** 1 Section for Wildlife Diseases, National Veterinary Institute, P.O. Box 8156 Dep., 0033 Oslo, Norway; 2 Section for Toxicology, National Veterinary Institute, P.O. Box 8156 Dep., 0033 Oslo, Norway; 3 Lillesand Veterinary Office, Ringgata 22A, 4790 Lillesand, Norway

## Abstract

Copper (Cu) deficiency was diagnosed in a Norwegian red deer (*Cervus elaphus*) herd subsequent to deaths due to emaciation in late autumn 1999. The animals had free access to salt licks containing 3000 mg Cu/kg. An evaluation of the herd revealed poor calf growth rate, low weights of adult hinds, dull and light-coloured hair coats and cases of diarrhoea. The herd was subsequently monitored throughout a three-year period of Cu-supplementation. The monitoring regimen included clinical observation, copper serum examination, weighing, faecal parasitological examination, and reproduction control by ultrasound. During the period January 2000 to May 2001, the animals were treated with Cu oxid capsules (1 g CuO/10 kg liveweight) at 2–4 months intervals, with the exception of March to September 2000. The animals were fed continuously with Cu-enriched concentrates containing 300 mg Cu/kg, at a rate of 1/2 kg per head and day, from May 2001 to January 2003. Following both copper supplementation regimens adequate serum Cu concentrations were measured, and markedly improved body weights, coat quality and reproductive results were observed, except for the period from March to September 2000 when no treatment was given. The results showed that in a deer herd, with a diet low in Cu, supplementation with CuO capsules had to be given at intervals of a few months to maintain adequate serum Cu levels. Free access to Cu-containing salt licks did not meet the animals' Cu demand. Good and stable results were achieved by the daily feeding of Cu-enriched concentrates.

## Findings

Copper (Cu) deficiency causes various disease syndromes in ruminants [1]. In farmed red deer (*Cervus elaphus*), deficiency has been associated with general unthrift, recognized as poor body condition, growth rates and coats [2-5], as well as enzootic ataxia [6,7] and osteochondrosis [8,9]. Another trace element deficiency observed in farmed deer is selenium (Se) deficiency, causing nutritional myopathy and Se responsive ill-thrift [10,11]. Clinical signs of Cu or Se deficiency have not been recorded in free-ranging red deer although low levels of both elements have been found in Norwegian populations [12]. This study presents clinical, chemical and reproductive data in a Cu-deficient Norwegian red deer herd that was diagnosed in late autumn 1999, and subsequently followed up throughout a three-year period of Cu-supplementation.

The herd was established in 1995, and consisted of approximately 15 adult hinds with additional calves and yearlings. The animals were kept in a 20 hectare enclosure consisting of permanent grazing land and deciduous forest. In summer, they also grazed cultivated ryegrass and white clover fields. During winter the herd was fed baled grass silage ad libitum and concentrates produced for dairy cows (105 neutral, Felleskjøpet, Norway) containing 20 mg Cu per kg, a ration of 1/2 kg per head per day. The animals also had free access to salt licks (KNZ Vilt, Felleskjøpet, Norway), containing 3000 mg Cu/kg. The herd was dewormed in the spring and autumn, using Ivomec pour-on vet.^® ^(Merial SAS, Lyon, France). The normal mating period was October with births in late May to early June.

During late autumn 1999, generally thin and unthrifty animals with dull light-coloured hair coats and cases of diarrhoea were observed. Three adult hinds died in an emaciated condition. Two of them were necropsied in the field. The third hind was necropsied in the laboratory, following standard procedures. Samples from the brain, spinal cord, lungs, heart, liver and kidneys were fixed in 10% buffered formalin, and processed routinely for histological examination. Standard bacteriological examination on calf blood agar plates was performed on samples of lung, liver and intestinal content. Additionally, specific bacteriological examination for *Mycobacterium avium subspecies paratuberculosis *was carried out on the intestinal content, and on jejunal and ileocaecal lymph nodes [13]. At necropsy, the hind was cachectic and weighed 60 kg. Other pathological findings included a moderate verminous pneumonia caused by *Dictyocaulus *sp., and fluid contents throughout the intestinal canal. No histopathological lesions were found in the organs, and all bacteriological examinations were negative with regard to pathogenic bacteria. Chemical examination of liver tissue from the carcass revealed Cu and Se concentrations of 1.8 μg/g and 0.15 μg/g wet weight (ww) respectively. In succession to these findings, three yearling stags were slaughtered, and their mean liver concentrations of Cu and Se were determined to be 2.4 μg/g (2.0–3.2) and 0.24 μg/g (0.21–0.27) respectively. The analyses were principally performed as described previously [14]. In farmed deer, Cu concentrations in the liver <4 μg/g wet weight (ww) and serum concentrations <0.3 μg/ml are considered to represent deficiency [5], whereas a deficiency level of Se has not been defined.

In the subsequent study period from January 2000 to January 2003, the animals were mechanically immobilized for blood (serum) sampling, weighing and Cu-supplementation at various time points (Table 1). Serum samples were obtained from all age classes and at least 75% of the animals in the herd, and the mean herd serum concentration of Cu was calculated for each sampling. The mean weights of calves and adult hinds were calculated separately for each weighing. During the period January 2000 to May 2001, the animals were treated with Cu six times. On five of these occasions they were administrated capsules containing 2.5 g Cu oxide, one capsule per 25 kg liveweight (1 g/10 kg). On the sixth occasion, they were treated with calcium copper edetate given subcutaneously at doses of 50 mg for calves, and 100 mg for yearlings and adults. From May 2001 to January 2003, the animals were fed continuously with Cu-enriched concentrates (300 mg Cu/kg) produced for deer (Tilskuddfôr Hjort, Felleskjøpet, Norway), a ration of 1/2 kg per head and day. The winter-feeding of concentrates produced for dairy cows ceased after starting Cu-enriched concentrates. Pregnancy of hinds >2 years was determined by ultrasound in January 2000, 2001, 2002 and 2003. Faecal samples for parasitological examination were collected quarterly throughout the years 1999–2002, each sampling including 4–8 animals from different age groups. The samples were analysed for parasite eggs and oocysts using sugar flotation, and for lungworm larvae after baermannisation.

**Table 1:** Copper status in a red deer herd. Mean and range of serum copper (μg/ml), mean and range of live weights (kg) of adult hinds and calves, and copper administration; by month 2000 to 2003.

Month	Serum copper [Range]	Weights of adult hinds [Range]	Weights of calves [Range]	Copper administration
January 2000	0.31 (N = 23) [<0.10–0.86]	82 (N = 12) [67–94]	38 (N = 7) [27–45]	x^1^
March 2000	0.84 (N = 27) [0.30–1.10]			x^2^
April 2000		90 (N = 13) [70–102]	48 (N = 7) [42–55]	
May 2000	0.92 (N = 23) [0.66–1.20]			
September 2000	0.25 (N = 20) [<0.10–0.62]			x^1^
November 2000				x^1^
January 2001	0.91 (N = 19) [0.30–2.00]	92 (N = 14) [72–102]	40 (N = 3) [38–45]	x^1^
May 2001				x^1,3^
September 2001	1.08 (N = 24) [0.70–1.66]			x^3^
January 2002	0.77 (N = 12) [0.61–0.99]	108 (N = 12) [94–126]	53 (N = 8) [51–58]	x^3^
May 2002	1.11 (N = 21) [0.78–1.60]			x^3^
September 2002	1.00 (N = 26) [0.34–1.50]			x^3^
January 2003		104 (N = 16) [81–120]	64 (N = 10) [57–74]	x^3^

x^1 ^Copper capsules; Copacaps^®^, Rhône Merieux Limitid, Harlow, Essex, UK.

x^2 ^Copper injectable; Coprin^®^, Mallinckrodt Veterinary Ltd, Breakspear Road South, Harefield, Middlesex, UK

x^3 ^From May 2001 the herd was fed continuously with concentrates containing 300 mg Cu per kg, 1/2 kg per head per day

The mean serum Cu concentrations of the animals in the herd at different time points from January 2000 to September 2002 are summarized in Table 1. The Cu concentration increased from a low level in January 2000 to an adequate level in March and May following the Cu-treatment in January and March. The mean serum Cu concentration, with no additional Cu treatments, again fell to a low level by September 2000. Levels returned to normal by January 2001 after Cu-treatment in September and November 2000. Following treatment in January and May 2001, and the daily feeding of Cu-enriched concentrates from May 2001, the mean serum Cu concentration of the herd appeared adequate when measured in September 2001, and in May and September 2002.

In January 2000, the weights of adult hinds and calves (Table 1) were low and ultrasound scans revealed only 23% as pregnant. Following Cu treatment in January and March 2000, markedly bettered body condition and hair coat of the animals were observed. The weight gain in adult hinds and calves from January to April 2000 was 10% and 26% respectively. Another remarkable feature that occurred following this Cu treatment was that several hinds came into oestrus out of season and were covered, resulting in calving in November and December 2000. Two fully developed newborn calves were found dead in the enclosure whilst a third calf was found alive in the moribund state. The weight gain of the animals during summer 2000 was, in spite of good grazing conditions on luxuriant cultivated pasture, not satisfactory and the mean weights of calves and adult hinds in January 2001 were only 5% and 11% respectively higher than those seen in January 2000. The percentage of hinds found to be pregnant in January 2001 was 46%. During the summers of 2001 and 2002 the weight gain in the herd was good, and the mean weights of calves and adult hinds in January 2002 and 2003 were 59% and 29% respectively above the level in January 2000. The reproductive success also improved remarkably, with 90% of hinds scanned in January 2002 and 2003 being pregnant. Faecal parasitological examinations of the animals in the herd revealed only low to moderate counts of parasite eggs and oocysts, and of lungworm larvae.

The findings in the herein reported red deer were consistent with unthrift related to a general Cu-deficiency in the herd, as reported in other studies [2-5]. Whether this deficiency was a result of an inadequate concentration of Cu in the diet (primary deficiency), or a result of dietary excess of antagonists (Mo, Fe, Zn, S), interfering with the utilization of Cu (secondary deficiency) [1], could not be determined. Specific syndromes of disease, i.e. enzootic ataxia and osteochondrosis, that have previously been reported in Cu-deficient red deer herds in Norway [9,15], were not observed. Whether the cases of diarrhoea seen in the present study were related to Cu-defiency remains an open question. However, diarrhoea of uncertain pathogenesis has been associated with Cu-deficiency in domestic ruminants [1].

The low rate of pregnancy observed in January 2000 was presumably an indirect effect of Cu deficiency, linked to the low body weights of hinds. No evidence of reproductive failure associated with specific Cu-mechanisms has been reported in domestic ruminants [1], and good nutritional status of deer hinds prior to mating is essential to achieve not only high fertility but also a concentrated calving season [16]. The Cu supplementation that was carried out in January and March 2000 resulted in general weight gain of hinds, and conception of several hinds in late spring, which is highly unusual. Other results of Cu supplementation seen in the present study included growth response in calves, and improved coat quality. These are all well known effects reported in several studies of Cu supplementation in deficient deer herds [3-5].

The liver concentrations of Se found in the red deer in the present study were above the minimum concentration considered to be adequate in domestic ruminants [1]. However, there are strong indications that both Cu and Se are trace elements of concern in Norwegian red deer farming. Deficiencies or marginal concentrations of both elements have been found in many farms having submitted liver samples for the control of trace element status to the National Veterinary Institute, and have also been demonstrated in fallow deer (*Dama dama*), moufflon (*Ovis aries musimon*) and moose (*Alces alces*) held in parks in this country (Unpublished data).

In the present study, the oral treatment with CuO capsules increased the Cu concentration in serum to within adequate levels, but only for a relatively short time-period. Between March and September 2000, when no capsules were given, the serum Cu concentration again dropped to inadequate levels. This should also be seen in connection with the use of cultivated grass pastures during summer; rapidly-growing grass is known to be poor in Cu [17]. This drop in serum Cu concentration during the main period of growth and weight gain over the summer presumably contributed to the low body weights found in January 2001. For maintenance of adequate serum levels with a diet low in Cu, it seemed that CuO capsules need to be given at intervals of a few months. This is in accordance with observations reported in other studies [3,18,19]. The free access of the herd to Cu-containing salt licks obviously did not meet the animals' Cu demand. Good and stable results were achieved by the daily feeding of Cu-enriched concentrates, used during the second part of this study.

## Competing interests

The authors declare that they have no competing interests.

## Authors' contributions

KH performed the post mortem examination, grouped the data, and drafted the manuscript. AB was responsible for the trace element analyses and results, and also contributed in writing the manuscript. MSA performed the clinical observations and collection of samples. The parasitological examination of faecal samples was carried at Section for Parasitology, Norwegian School of Veterinary Science. All authors read and approved the final manuscript.
